# Supplementary material for: Fire risk to structures in California’s Wildland-Urban Interface
Source: Nat Commun. 2025 Aug 28;16:8041. doi: 10.1038/s41467-025-63386-2 (PMC12394485; doi:10.1038/s41467-025-63386-2)
Supplement: Supplementary file 1 — Supplementary Information [file 41467_2025_63386_MOESM1_ESM.pdf]

Fire Risk to Structures in California's Wildland-Urban Interface

Supplementary Materials

We employed several classification models to analyze the Stacked WUI data and individual fires. In this section, we present the results of the Logistic Regression, Random Forest, and CatBoost models applied to the Stacked WUI data and individual fires. Each of these models provides unique advantages and approaches for evaluating feature contributions through SHAP values. The results of the Logistic Regression (Supplementary Fig.1, Supplementary Table 1), Random Forest (Supplementary Fig.2, Supplementary Table 2), and the CatBoost (Supplementary Fig.3, Supplementary Table 3) are shown below.

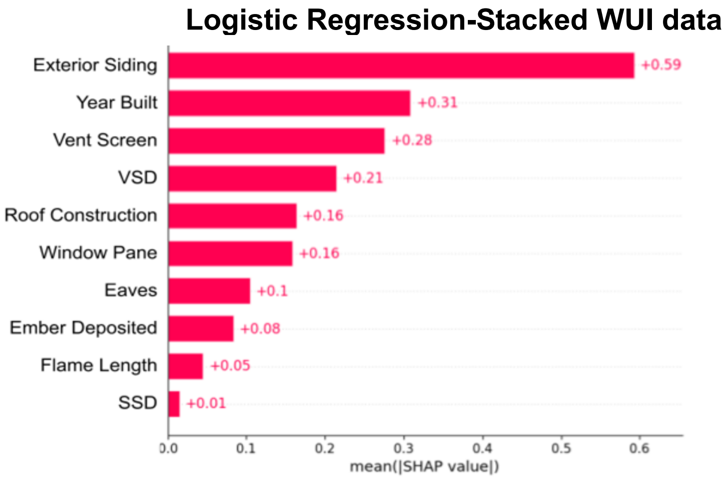

Logistic Regression-Individual Fires

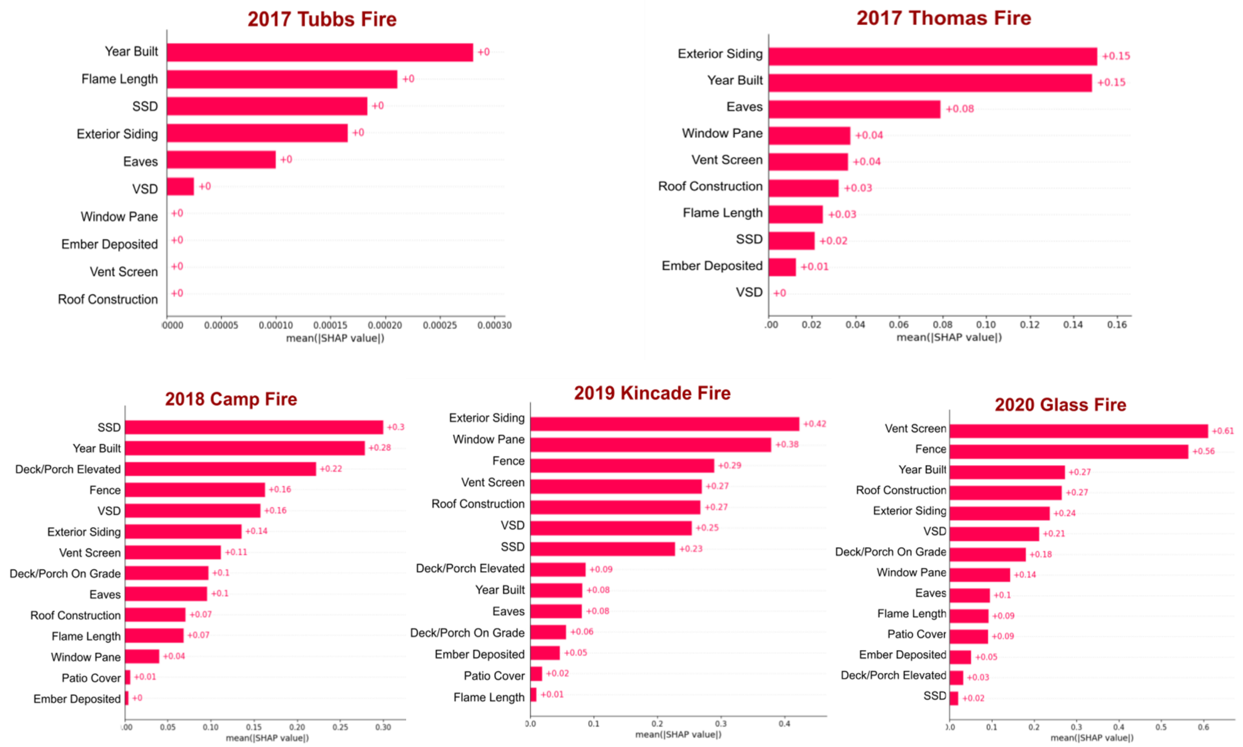

Supplementary Fig.1- SHAP results with Logistic Regression; n = 47,742 (Stacked WUI data); n = 13,027 (Tubbs); n = 5,192 (Thomas); n = 23,204 (Camp); n = 1,555 (Kincade); n = 4,768 (Glass).

Supplementary Table 1- Results of Logistic Regression predictions on each test set with a resulting metrics displaying model performance including area under an ROC curve (AUC), and the percentage of correct predictions the model makes (Accuracy), the accuracy of positive predictions (Precision), model's ability to identify all positive instances (Recall), and the harmonic mean of precision and recall (F1 Score).

WUI Fire	AUC	Accuracy	Precision	Recall	F1-Score
Tubbs	0.54	0.94	0.94	1	0.97
Thomas	0.64	0.75	0.75	1	0.85
Camp	0.75	0.82	0.82	0.98	0.89
Kincade	0.58	0.75	0.41	0.24	0.31
Glass	0.78	0.75	0.67	0.45	0.54
5 Fires Combined	0.65	0.78	0.82	0.95	0.88
All CA DINS (2017-22)	0.81	0.75	0.77	0.82	0.79

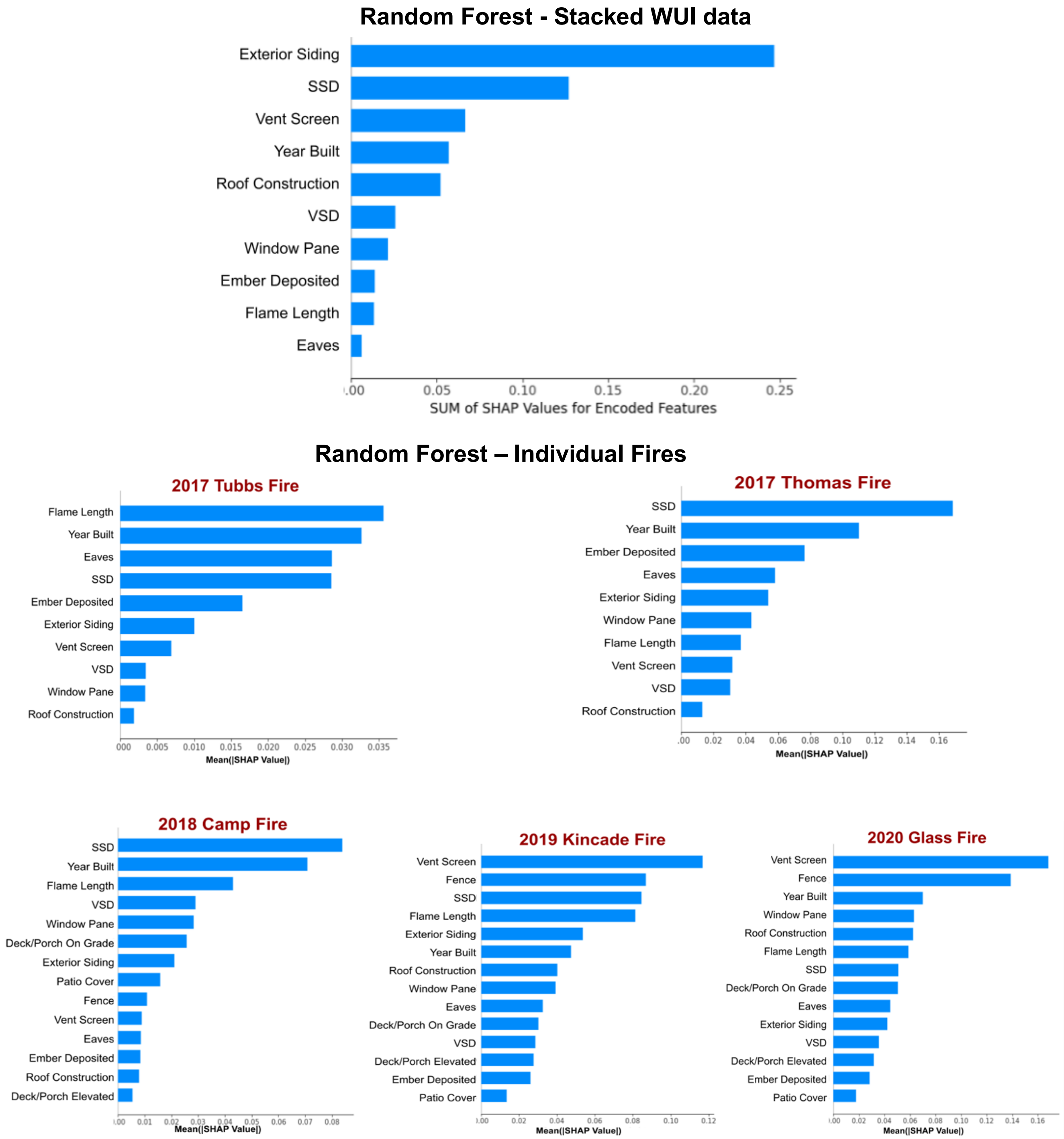

Supplementary Fig.2- SHAP results with Random Forest; n = 47,742 (Stacked WUI data); n = 13,027 (Tubbs); n = 5,192 (Thomas); n = 23,204 (Camp); n = 1,555 (Kincade); n = 4,768 (Glass).

Supplementary Table 2- Results of Random Forest predictions on each test set with a resulting metrics displaying model performance including area under an ROC curve (AUC), and the percentage of correct predictions the model makes (Accuracy), the accuracy of positive predictions (Precision), model's ability to identify all positive instances (Recall), and the harmonic mean of precision and recall (F1 Score).

WUI Fire	AUC	Accuracy	Precision	Recall	F1-Score
Tubbs	0.71	0.94	0.94	1	0.97
Thomas	0.81	0.81	0.84	0.92	0.88
Camp	0.78	0.81	0.81	0.99	0.89
Kincade	0.71	0.66	0.36	0.62	0.45
Glass	0.84	0.79	0.85	0.43	0.57
5 Fires Combined	0.83	0.81	0.84	0.94	0.89
All CA DINS (2017-22)	0.82	0.75	0.78	0.81	0.79

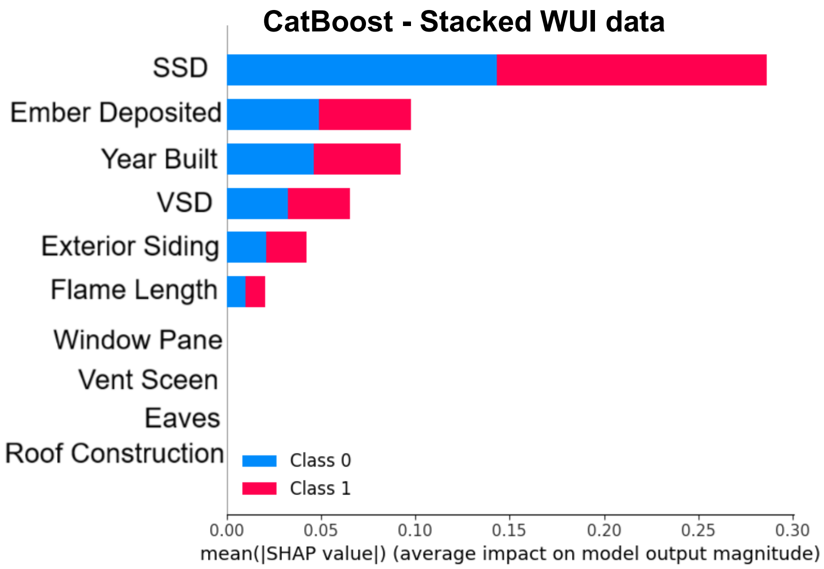

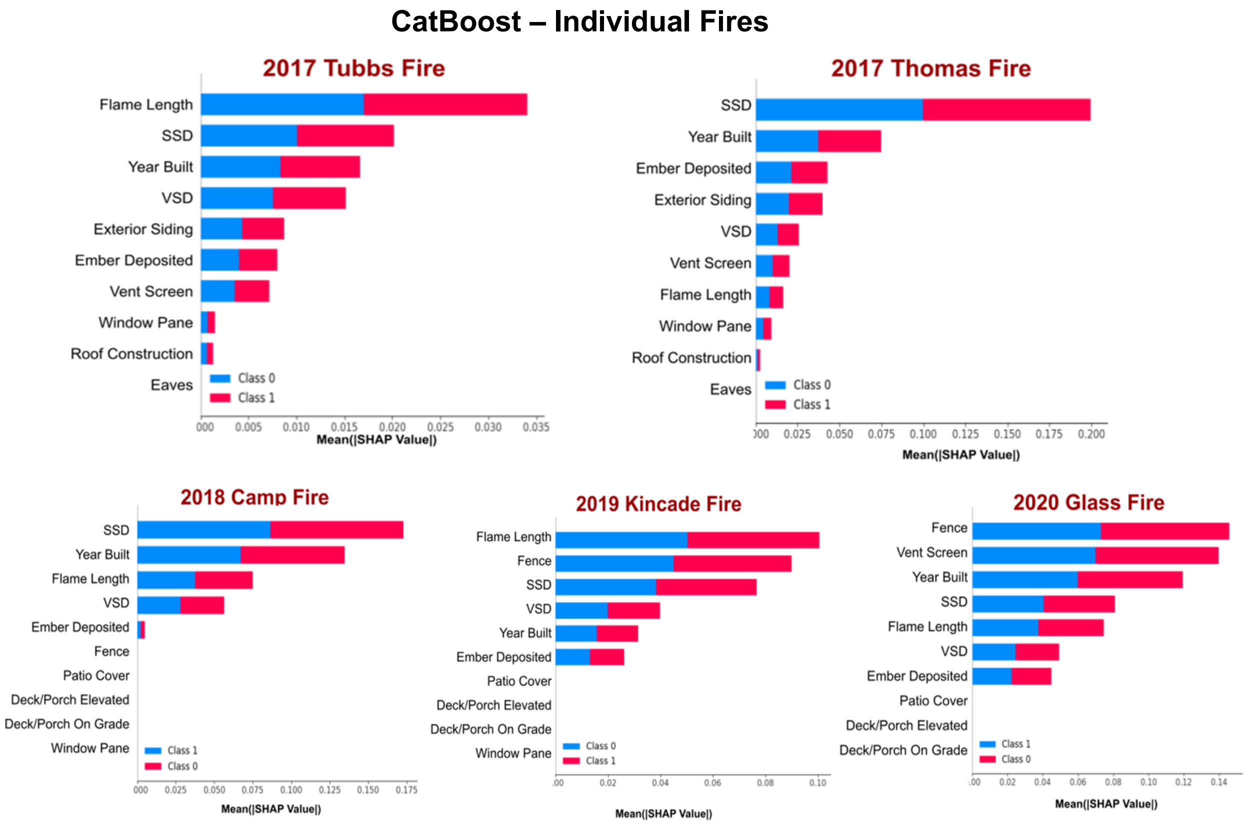

Supplementary Fig.3- SHAP results with CatBoost (Class 0= Survived, Class 1= Destroyed); n = 47,742 (Stacked WUI data); n = 13,027 (Tubbs); n = 5,192 (Thomas); n = 23,204 (Camp); n = 1,555 (Kincade); n = 4,768 (Glass).

Supplementary Table 3- Results of CatBoost predictions on each test set with a resulting metrics displaying model performance including area under an ROC curve (AUC), and the percentage of correct predictions the model makes (Accuracy), the accuracy of positive predictions (Precision), model's ability to identify all positive instances (Recall), and the harmonic mean of precision and recall (F1 Score).

WUI Fire	AUC	Accuracy	Precision	Recall	F1-Score
Tubbs	0.59	0.94	0.89	0.94	0.92
Thomas	0.73	0.77	0.82	0.77	0.70
Camp	0.74	0.81	0.78	0.81	0.76
Kincade	0.65	0.77	0.59	0.77	0.67
Glass	0.73	0.76	0.79	0.76	0.71
5 Fires Combined	0.80	0.80	0.78	0.80	0.77
All CA DINS (2017-22)	0.80	0.75	0.75	0.75	0.75

The ROC Curves and Confusion Matrices for the XGBoost model applied to the five fires are shown in Supplementary Fig.4. The ROC Curves provide insight into the model's ability to discriminate between different classes, with the area under the curve (AUC) serving as a measure of its overall performance. The Confusion Matrices offer a detailed breakdown of the model's predictions, highlighting the number of true positives, false positives, true negatives, and false negatives for each fire scenario.

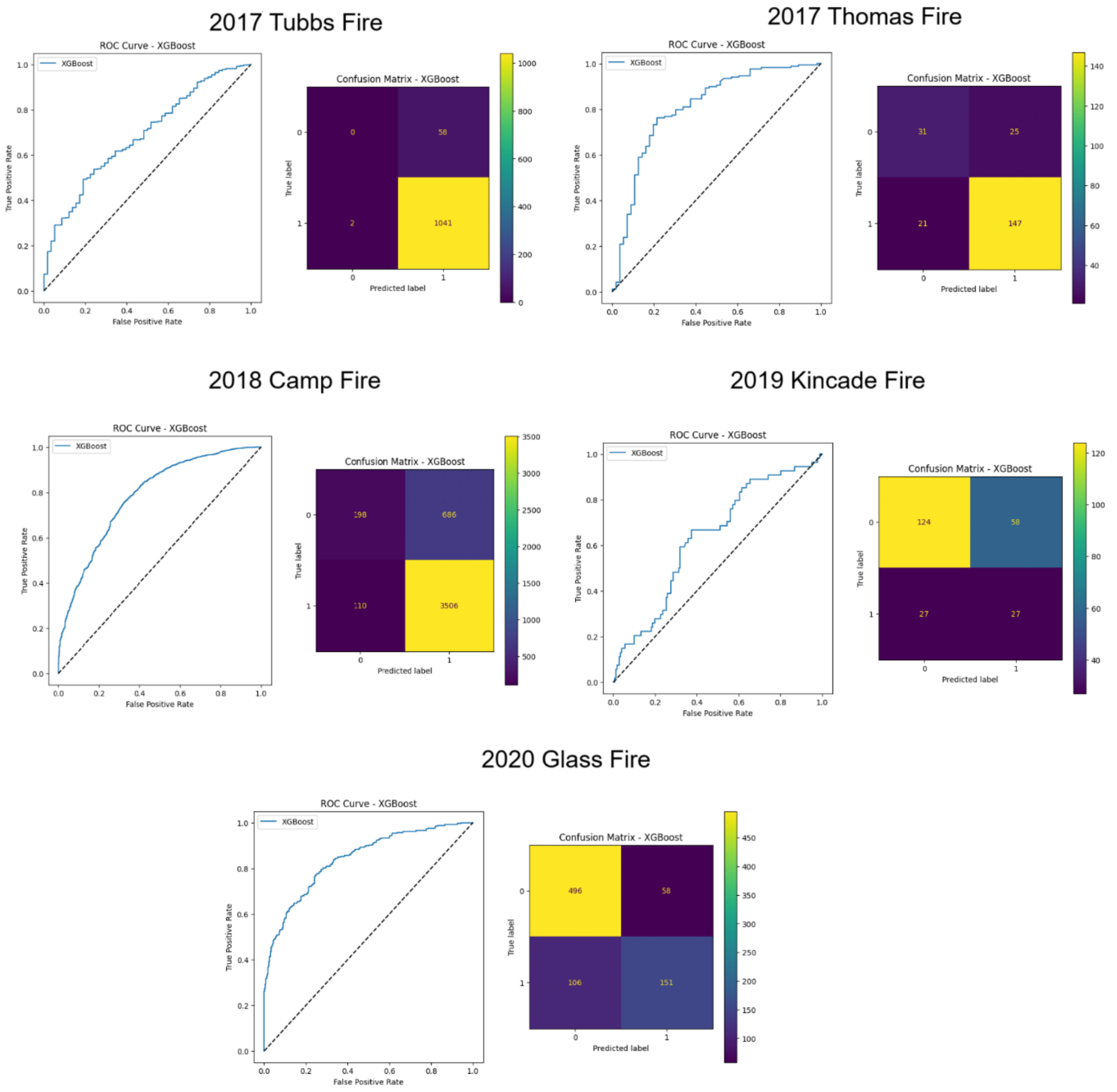

Supplementary Fig.4- ROC Curves and Confusion Matrices for the XGBoost model and five fires; n = 13,027 (Tubbs); n = 5,192 (Thomas); n = 23,204 (Camp); n = 1,555 (Kincade); n = 4,768 (Glass).

Supplementary Figs.5-9 illustrate the results of different mitigation strategies tested using our model, which examines the impact of home hardening and defensible space clearing on structure loss (Class 1) and survivability (Class 0). In the No-Mitigation Scenario (Supplementary Fig.5),

structure loss is 80%, with a survivability rate of 20%. In Scenario 1 (Supplementary Fig.6), home hardening measures, including non-flammable siding, fine mesh over vents, and non-flammable roofs, were applied. This resulted in a 25% survival rate and 75% structure loss. Scenario 2 (Supplementary Fig.7) combined home hardening with clearing defensible space in Zone 0 (0-5 feet), which improved the survival rate to 40% and reduced structure loss to 60%. In the Zone 0-only clearance scenario (Supplementary Fig. 8), survival reached 37 %, with a 63 % loss. Finally, in the extreme mitigation scenario (Supplementary Fig.9), both home hardening and defensible space clearing in Zones 0 (0-5 feet) and 1 (5-30 feet) were implemented, leading to a 48% survival rate and a 52% reduction in structure loss.

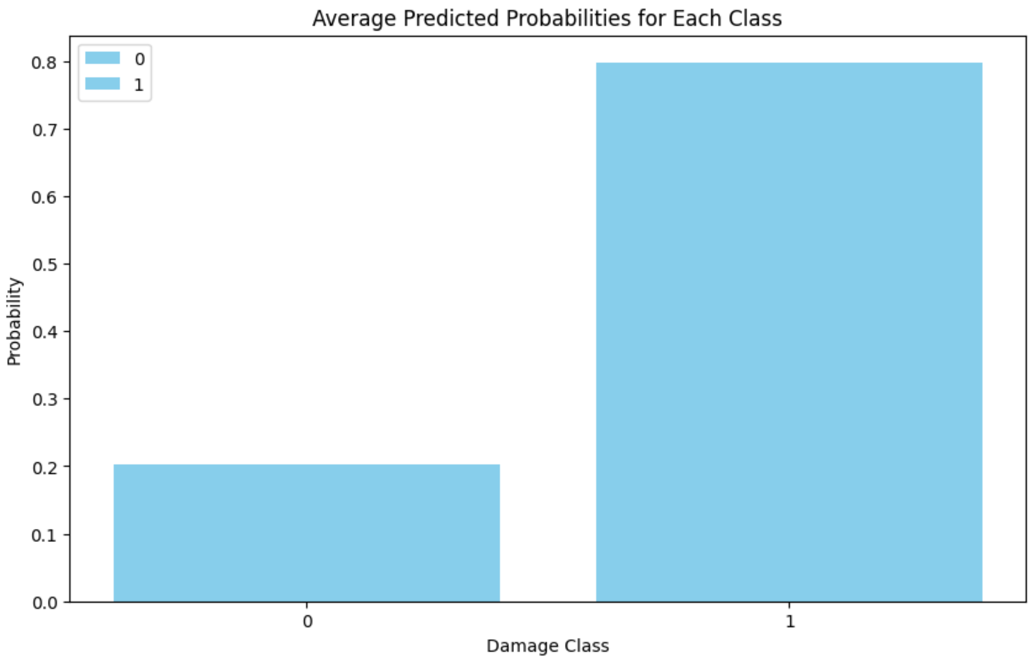

Supplementary Fig.5- Predicting probabilities of structural destruction for entire WUI data (n=47,742)

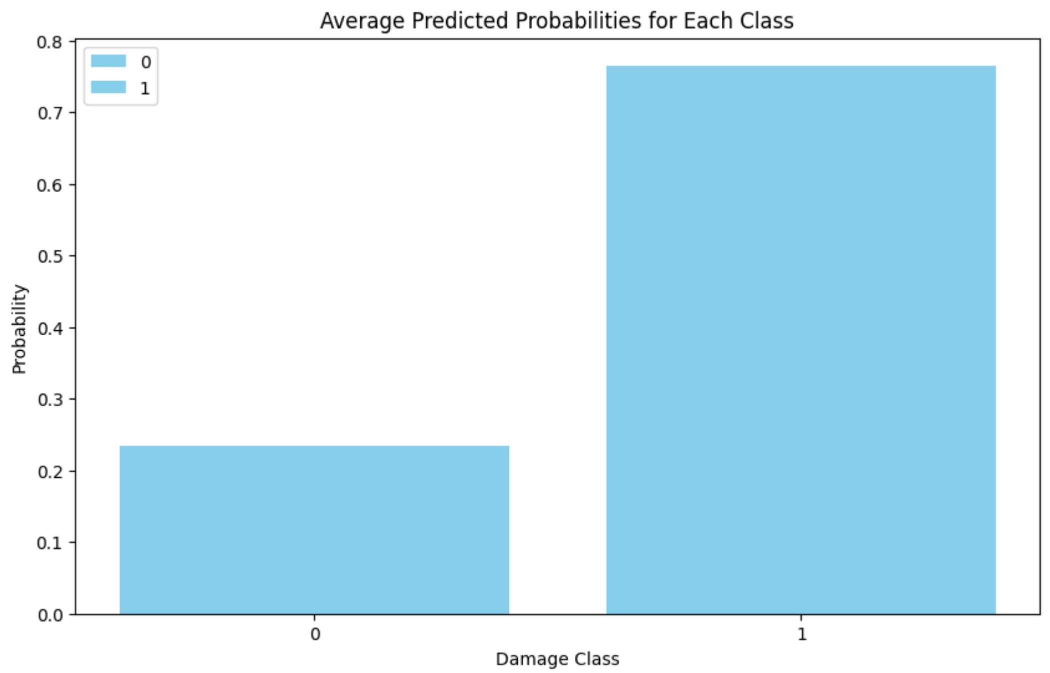

Supplementary Fig.6- Predicting probabilities of structural destruction with home hardening scenario (n=47,742)

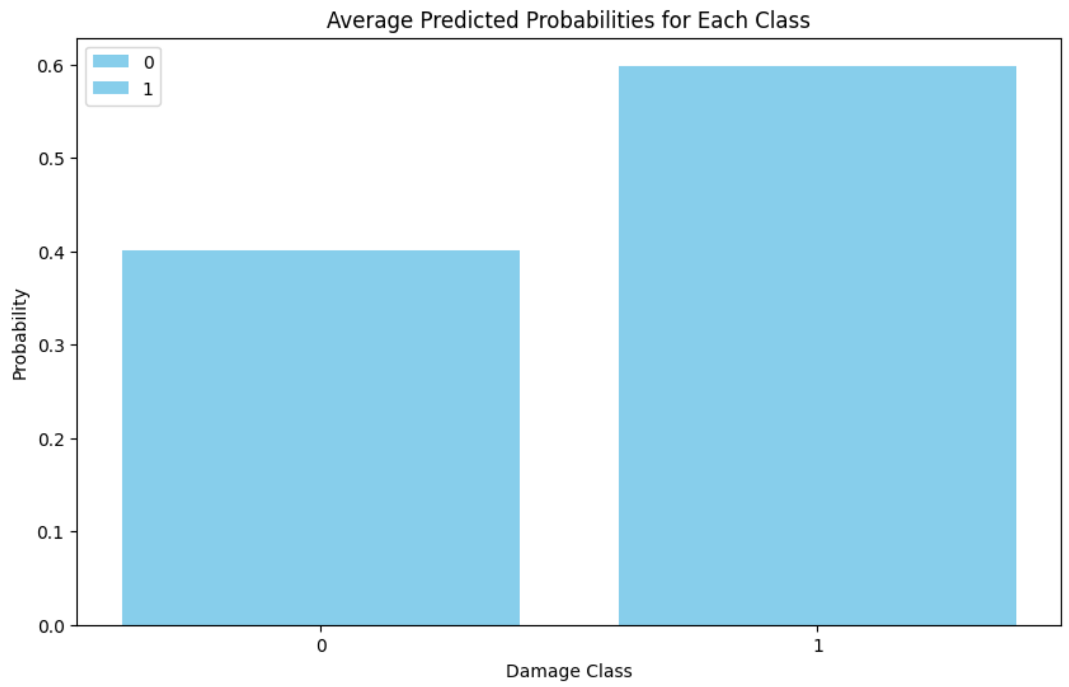

Supplementary Fig.7- Predicting probabilities of structural destruction with home hardening and defensible space (clearing Zone 0) (n=47,742)

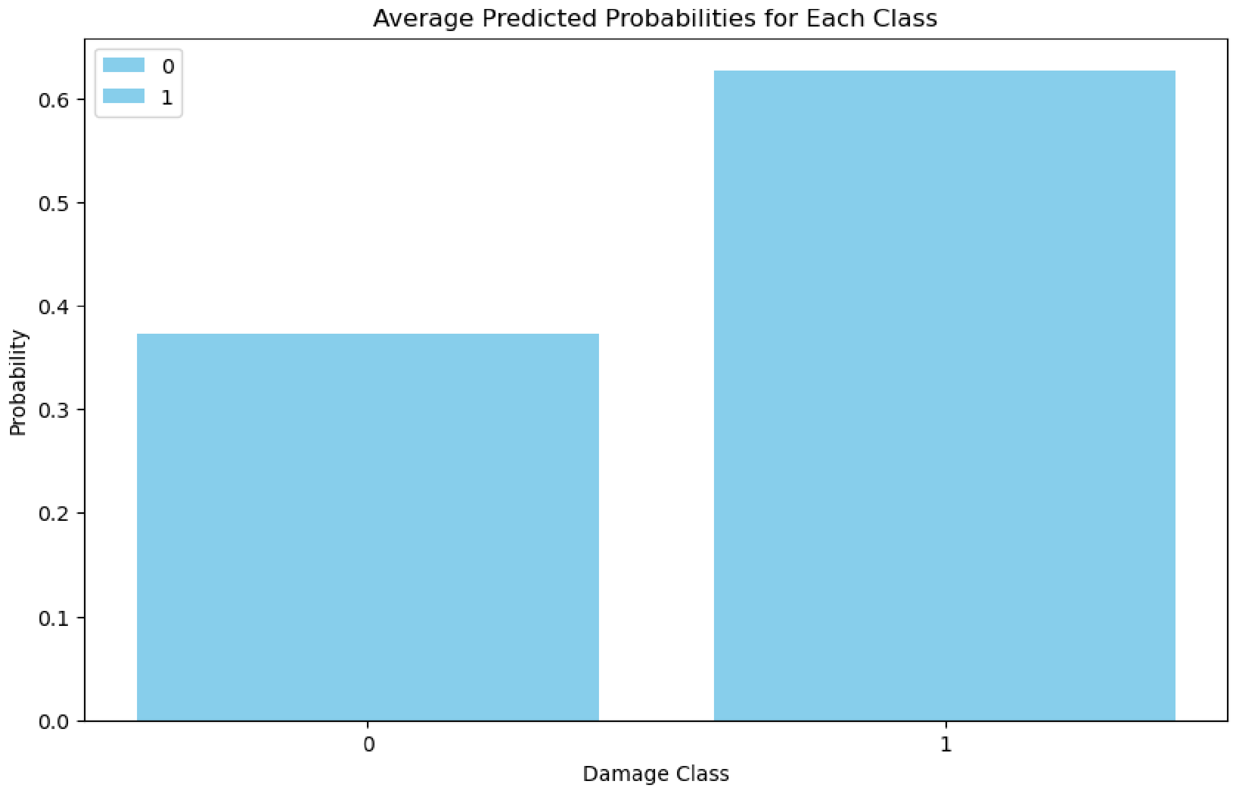

Supplementary Fig.8- Predicting probabilities of structural destruction with applying defensible space (clearing Zone 0) (n=47,742)

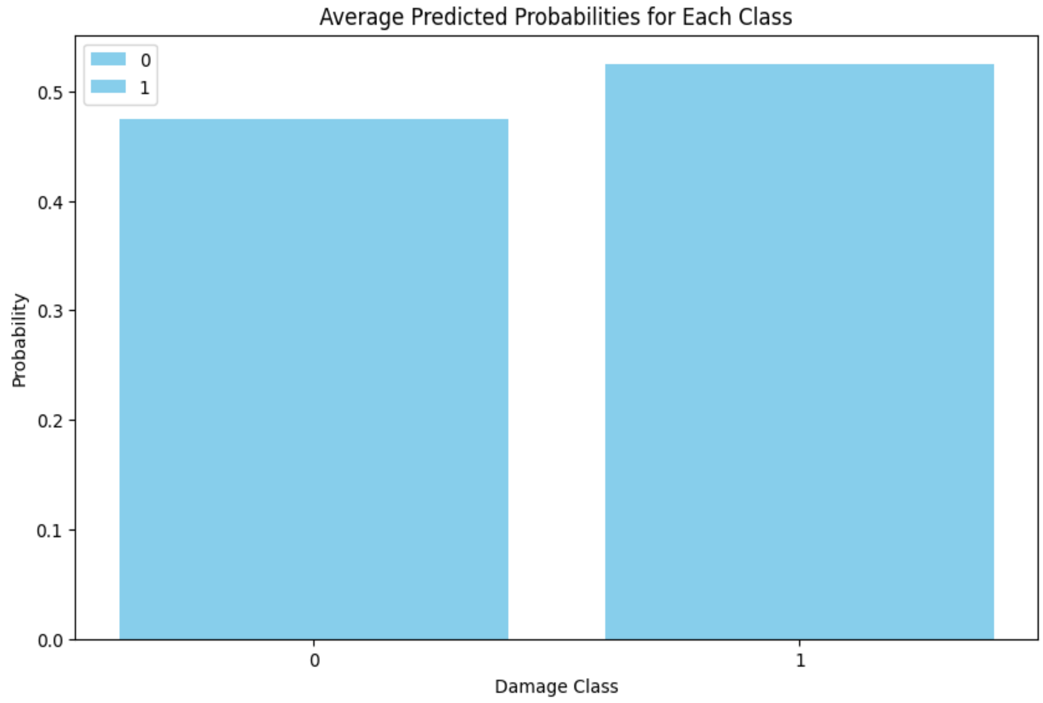

Supplementary Fig.9- Predicting probabilities of structural destruction with home hardening and defensible space (clearing Zone 0 and Zone 1) (n=47,742)
