# Supplementary material for: Fire risk to structures in California’s Wildland-Urban Interface
Source: Nat Commun. 2025 Aug 28;16:8041. doi: 10.1038/s41467-025-63386-2 (PMC12394485; doi:10.1038/s41467-025-63386-2)
Supplement: Supplementary file 2 — Reporting Summary [file 41467_2025_63386_MOESM2_ESM.pdf]

nature portfolio

Corresponding author(s): Michael Gollner

Last updated by author(s): Jul 1, 2025

# Reporting Summary

Nature Portfolio wishes to improve the reproducibility of the work that we publish. This form provides structure for consistency and transparency in reporting. For further information on Nature Portfolio policies, see our Editorial Policies and the Editorial Policy Checklist.

## Statistics

For all statistical analyses, confirm that the following items are present in the figure legend, table legend, main text, or Methods section.

| n/a | Confirmed | |
|---|---|---|
| ☐ | ☒ | The exact sample size (*n*) for each experimental group/condition, given as a discrete number and unit of measurement |
| ☐ | ☒ | A statement on whether measurements were taken from distinct samples or whether the same sample was measured repeatedly |
| ☐ | ☒ | The statistical test(s) used AND whether they are one- or two-sided<br>*Only common tests should be described solely by name; describe more complex techniques in the Methods section.* |
| ☐ | ☒ | A description of all covariates tested |
| ☐ | ☒ | A description of any assumptions or corrections, such as tests of normality and adjustment for multiple comparisons |
| ☐ | ☒ | A full description of the statistical parameters including central tendency (e.g. means) or other basic estimates (e.g. regression coefficient) AND variation (e.g. standard deviation) or associated estimates of uncertainty (e.g. confidence intervals) |
| ☒ | ☐ | For null hypothesis testing, the test statistic (e.g. *F*, *t*, *r*) with confidence intervals, effect sizes, degrees of freedom and *P* value noted<br>*Give P values as exact values whenever suitable.* |
| ☒ | ☐ | For Bayesian analysis, information on the choice of priors and Markov chain Monte Carlo settings |
| ☒ | ☐ | For hierarchical and complex designs, identification of the appropriate level for tests and full reporting of outcomes |
| ☒ | ☐ | Estimates of effect sizes (e.g. Cohen's *d*, Pearson's *r*), indicating how they were calculated |

*Our web collection on statistics for biologists contains articles on many of the points above.*

## Software and code

Policy information about availability of computer code

**Data collection**

• CAL FIRE DINS damage records (2013–2022) for ~90 k structures, curated to ~47 k across five fires. all derived geospatial and modeling outputs are deposited in the DINS_data_analysis repository: https://github.com/berkeley-firelab/DINS_data_analysis/data
• Microsoft US Building Footprints (2019–2020) for structure density (SSD): https://github.com/microsoft/USBuildingFootprints
• Sonoma County LiDAR (2013–2017) and fine-scale vegetation map for defensible space/ Vegetation Separation Distance (VSD) analysis: LiDAR: https://sonomavegmap.org/
• Google Earth Pro & NAIP imagery (2016–2019) for vegetation classification

**Data analysis**

• Programming Language: Python (v3.10.8)
• Data Manipulation & Analysis: pandas (v1.5.3), numpy (v1.24.2)
• Machine Learning & Statistical Modeling: Logistic Regression & Random Forest scikit-learn (v1.2.1), XGBoost (v1.7.5), CatBoost (v1.1.1)
• Model Interpretation: shap (v0.41.0) (https://shap.readthedocs.io/en/latest/)
• Geospatial Analysis: geopandas (v0.12.2), rasterio (v1.3.0), QGIS (v3.28.1)
• Fire Spread Simulation: ELMFIRE (Eulerian Level Set Model of FIRE) with Monte Carlo ensembles for flame length and ember load estimation (https://github.com/lautenberger/elmfire)
• Earth Engine API: earthengine-api (v0.1.362)
• Imputation & Preprocessing: scikit-learn IterativeImputer (k-NN & median strategies), StandardScaler, OneHotEncoder, LabelEncoder, Stratified train/test split (80/20) preserving damage-class proportions
• Hyperparameter Tuning & Validation: scikit-learn GridSearchCV, RandomizedSearchCV (cv=10)
• Visualization: matplotlib (v3.6.2)
• Spatial Distance Calculations: custom spatial-clustering and distance routines (Haversine & pairwise in UTM) for geographically aware

imputation

For manuscripts utilizing custom algorithms or software that are central to the research but not yet described in published literature, software must be made available to editors and reviewers. We strongly encourage code deposition in a community repository (e.g. GitHub). See the Nature Portfolio guidelines for submitting code & software for further information.

## Data

Policy information about availability of data

All manuscripts must include a data availability statement. This statement should provide the following information, where applicable:

- Accession codes, unique identifiers, or web links for publicly available datasets
- A description of any restrictions on data availability
- For clinical datasets or third party data, please ensure that the statement adheres to our policy

The datasets generated during and/or analyzed during the current study are available in the [DINS_data_analysis] repository, [https://github.com/berkeley-firelab/DINS_data_analysis]; [https://doi.org/10.5281/zenodo.15776778]63.

## Research involving human participants, their data, or biological material

Policy information about studies with human participants or human data. See also policy information about sex, gender (identity/presentation), and sexual orientation and race, ethnicity and racism.

| | |
|---|---|
| Reporting on sex and gender | N/A |
| Reporting on race, ethnicity, or other socially relevant groupings | N/A |
| Population characteristics | N/A |
| Recruitment | N/A |
| Ethics oversight | N/A |

Note that full information on the approval of the study protocol must also be provided in the manuscript.

# Field-specific reporting

Please select the one below that is the best fit for your research. If you are not sure, read the appropriate sections before making your selection.

☐ Life sciences ☐ Behavioural & social sciences ☒ Ecological, evolutionary & environmental sciences

For a reference copy of the document with all sections, see nature.com/documents/nr-reporting-summary-flat.pdf

# Ecological, evolutionary & environmental sciences study design

All studies must disclose on these points even when the disclosure is negative.

| | |
|---|---|
| Study description | We combined records of damage state and building features from the DINS dataset with remotely-sensed assessment of surrounding vegetation (akin to defensible space) and structure footprints (to assess building separation) of undamaged, damaged, and destroyed structures within the final fire perimeter (CAL FIRE Historic Fire Perimeters), including a 91 m (300 ft) buffer around any burned areas. Post-fire reconstruction modeling was then used to add local fire exposure by both flames (flame length) and embers (ember load) to the dataset resulting in a more complete picture of fire exposure and effects. |
| Research sample | Structures inspected by CAL FIRE crews (DINS program) following 2013–2022 wildfires; selected five major WUI events (2017 Tubbs, 2017 Thomas, 2018 Camp, 2019 Kincade, 2020 Glass) based on data completeness and number of exposed structures. |
| Sampling strategy | • Extracted structures within CAL FIRE Historic Fire Perimeters plus a 91m buffer using GIS overlays.<br>• Supplemented with geospatial and remotely sensed data (Structure polygons, LiDAR, imagery) and fire exposure outputs (fire spread model). |
| Data collection | • Damage Inspections: Conducted immediately post-fire between 2013 and 2022. Recorded by CAL FIRE inspection crews using standardized DINS forms and mobile GIS tools.<br>• Vegetation & Structure Data: Use open source and publicly available data from 2013–2017 (LiDAR: https://sonomavegmap.org/ ), 2016–2019 aerial and street-level imagery from Google Earth Pro and NAIP for vegetation mapping, and Microsoft's US Building Footprints from 2019–2020 (https://github.com/microsoft/USBuildingFootprints).<br>• Fire Exposure Modeling: Conducted with ELMFIRE simulations, incorporating urban spread via empirical HAMADA extension (https://github.com/lautenberger/elmfire). |

| | |
|---|---|
| Timing and spatial scale | • Spatial Resolution: Structure-level analysis within fire perimeters + 91m buffer, 1m airborne LiDAR data and 30x30 m grid for fuel data from LANDFIRE.<br>Timing: : Structures inspected by CAL FIRE crews (DINS program) following 2013–2022 wildfires; selected five major WUI events (2017 Tubbs, 2017 Thomas, 2018 Camp, 2019 Kincade, 2020 Glass) based on data completeness and number of exposed structures. |
| Data exclusions | No pre-established exclusion criteria. All structures with valid DINS records were included. Missing exposure and vegetation values were imputed per the described methods. |
| Reproducibility | Replication not applicable; observational retrospective design using complete available dataset. |
| Randomization | N/A |
| Blinding | • Not applicable; data collection and analysis were not blinded. |

Did the study involve field work? ☐ Yes ☒ No

# Reporting for specific materials, systems and methods

We require information from authors about some types of materials, experimental systems and methods used in many studies. Here, indicate whether each material, system or method listed is relevant to your study. If you are not sure if a list item applies to your research, read the appropriate section before selecting a response.

## Materials & experimental systems

| n/a | Involved in the study |
|---|---|
| ☒ | ☐ Antibodies |
| ☒ | ☐ Eukaryotic cell lines |
| ☒ | ☐ Palaeontology and archaeology |
| ☒ | ☐ Animals and other organisms |
| ☒ | ☐ Clinical data |
| ☒ | ☐ Dual use research of concern |
| ☒ | ☐ Plants |

## Methods

| n/a | Involved in the study |
|---|---|
| ☒ | ☐ ChIP-seq |
| ☒ | ☐ Flow cytometry |
| ☒ | ☐ MRI-based neuroimaging |

# Plants

| | |
|---|---|
| Seed stocks | N/A |
| Novel plant genotypes | N/A |
| Authentication | N/A |
